# Post COVID-19 vaccination headache: A clinical and epidemiological evaluation

**DOI:** 10.3389/fpain.2022.994140

**Published:** 2022-11-08

**Authors:** Giulia Ceccardi, Francesca Schiano di Cola, Marco Di Cesare, Paolo Liberini, Mauro Magoni, Cristiano Perani, Roberto Gasparotti, Renata Rao, Alessandro Padovani

**Affiliations:** ^1^ Neurology Unit, Department of Clinical and Experimental Sciences, University of Brescia, Brescia, Italy; ^2^ Neurology Unit, Department of Neurological and Vision Sciences, ASST Spedali Civili, Brescia, Italy; ^3^ Stroke Unit, Department of Neurological and Vision Sciences, ASST Spedali Civili, Brescia, Italy; ^4^ Emergency Department, ASST Spedali Civili, Brescia, Italy; ^5^ Neuroradiology Unit, Department of Medical and Surgical Specialties, Radiological Sciences and Public Health, University of Brescia and ASST Spedali Civili, Brescia, Italy

**Keywords:** COVID-19, vaccination, headache, SARS-CoV-2, migraine, trigeminovascular complex

## Abstract

**Introduction:**

This study aimed to assess the prevalence and clinical characteristics of headaches, in particular secondary headaches.

**Materials and Methods:**

This observational study was performed at the ASST Spedali Civili of Brescia, Italy. Visits to the Emergency Department (ED) and subsequent hospitalizations regarding a new or worsening headache in the 16 days following the administration of the COVID-19 vaccine between January 2021 and January 2022 were recorded and compared with those of January 2019–January 2020.

**Results:**

The ratio between ED admissions due to headaches and total ED admissions was significantly higher in 2021 compared with 2019 (4.84% vs. 4.27%; *p* < 0.0001). Two-hundred and eighty-nine ED headache admissions (10.8% of all ED headache admissions) were time-correlated to the COVID-19 vaccination, of which 40 were hospitalized in order to exclude a symptomatic etiology. At discharge, 32 patients had a diagnosis of benign headache not attributed to any cranial/extracranial disorder and eight patients of secondary headache, whose diagnoses were the following: Headache attributed to cranial and/or cervical vascular disorder (*n* = 4); headache attributed to nonvascular intracranial disorder (*n* = 2); headache or facial pain attributed to disorder of the cranium, neck, eyes, ears, nose, sinuses, teeth, mouth, or other facial or cervical structure (*n* = 1); and painful lesions of the cranial nerves (*n* = 1). The headache most frequently reported by patients had migraine-like characteristics: the localization was predominantly frontal or temporal, the pain was described as throbbing and severe in intensity and it was frequently accompanied by nausea/vomit, and photo-phonophobia. Over half—regardless of the final diagnosis—of hospitalized patients had a history of primary headaches.

**Conclusions:**

Following the spread of COVID-19 vaccination, the number of ED admissions due to headaches significantly increased. However, less than 14% of all the ED visits due to a headache time-correlated to the COVID-19 vaccination were actually hospitalized, with most patients documenting a benign headache, possibly related to the generic side effects of the vaccination. Only 8/40 hospitalized patients were diagnosed with a secondary headache. These benign headaches would actually fulfill diagnostic criteria for 8.1 Headaches attributed to the use of or exposure to a substance (ICHD-3), although, at the time being, it does not include vaccines as possible substances.

The headache migraine-like characteristics’ reported by most patients could suggest activation of the trigeminovascular pathway by all the cytokines and other pro-inflammatory molecules released following the vaccination.

## Introduction

Since December 2020, the Agenzia Italiana del Farmaco (AIFA) has authorized the release of COVID-19 vaccines (Comirnaty, Spikevax, Vaxzevria e Janssen) in Italy. Various adverse reactions have been reported and important population concerns about vaccine safety have emerged.

Nonetheless, the most frequent adverse reactions, reported for all types of vaccines, were local reactions, myalgia, asthenia, fever, and headache.

With regard to headache, this was reported in a percentage between 25% and 58%, with a greater frequency in subjects aged <55 years. Thus, the headache was the single neurological most frequent adverse reaction (1–4).

From the annual COVID-19 Vaccine Surveillance Report (27 December 2020 to 26 December 2021) (5), the trend of the adverse reactions’ reports and the relative rates are substantially stable over time, with a slight decline during the summer period and in concurrence with the second and/or third doses.

The general reporting rate is 109 reports for every 100,000 doses administered for a total of 117.920 reports, of which 83.7% are classified as nonserious and with a complete resolution.

Neurological adverse events, mainly due to the high prevalence of headaches, are the second most common adverse reaction. Severe neurological events, such as cerebral venous thrombosis (CVT), acute cerebrovascular events, subdural hematomas, Bell's palsy, seizures, and acute inflammatory demyelinating polyneuropathies are very rare.

Albeit these severe neurological events were a minority of vaccination side effects, during 2021, cases of CVTs after vaccination led to official measures to limit viral vector vaccines for some population categories, with major concerns regarding the vaccination campaign (5).

This complication is unfortunately unpredictable, but it can be clinically suspected and treated because its pathogenesis is mostly known. In adenovirus-based vaccines, the viral DNA binds to platelet factor 4 (PF4) creating a new antigen. Some predisposing patients, for an unknown mechanism, create anti-PF4 immunoglobulins, which activate and aggregate platelets, as observed in heparin-induced thrombocytopenia. Moreover, these antibodies stimulate tissue factor that, together with the activation of immune response mediated by vaccination, promotes coagulation cascade leading to thrombosis (6). This mechanism gives rise to vaccine-induced thrombotic thrombocytopenia (VITT) characterized by severe thrombocytopenia with atypical thrombosis (such as cerebral or splanchnic), which can be treated with anticoagulation and high-dose intravenous immunoglobulin (7, 8).

This study aimed to evaluate the prevalence, etiology, and clinical characteristics of secondary headaches following COVID-19 vaccination and to characterize post-COVID-19 vaccination headaches in general.

## Materials and methods

### Standard protocol approvals and patient consent

This study was approved by the ethics standards committee on human experimentation (local ethics committee of the ASST Spedali Civili Hospital, Brescia: Committee protocol number 0061646/21) on 3 August 2021.

### Study design and participants

The present observational study was conducted at the Neurology Unit and Emergency Department (ED) of the Spedali Civili Hospital of Brescia from January 2021 to January 2022.

The primary objective was to evaluate the number of ED admissions and neurological hospitalizations due to headaches with onset or worsening after the COVID-19 vaccine and to evaluate their etiology and clinical characteristics. The decision to hospitalize the patient was made based on the presence of well-established red flags (SNNOOP10 list) (9).

All patients admitted to the ED were initially screened. Inclusion criteria were the following: admission to the ED and/or subsequent Neurology hospitalizations regarding a new or worsening headache in the 16 days following the administration of the COVID-19 vaccine; the ability to provide informed consent; age ≥15 years. Patients were clinically assessed at the first visit to the ED and during the hospitalization. The period of 16 days was based on the finding that most thrombotic complications of COVID-19 vaccination were reported within the first 2 weeks following the vaccination (7, 8).

For patients discharged from the ED, available data consisted of clinical and/or neurological evaluation at the time of the ED presentation.

For hospitalized patients, information regarding headache characteristics, clinical history, clinical course during hospitalization, diagnostic-instrumental tests, inpatient pharmacological treatments, and the diagnosis at discharge were collected.

### Outcome measures

The primary endpoint was to assess the number of admissions and the etiology of new or worsening headaches within 16 days after the administration of COVID-19 vaccines. In particular, the number of admissions and hospitalizations in the pre-COVID-19 era (January 2019–January 2020) vs. the COVID-19 vaccination period (January 2021–January 2022) were collected and compared.

Furthermore, the number of hospitalizations for new or worsening headaches following the COVID-19 vaccine was compared with the number of hospitalizations for headaches not associated with the COVID-19 vaccine.

Secondary endpoints were: the identification of headache characteristics (type of pain, localization, pain intensity, associated neurological and/or systemic symptoms, initial clinical suspicion, diagnosis at discharge); the clinical course during hospitalization; and the identification of possible clinical and biohumoral markers, which could predict symptomatic headaches and the type of COVID-19 vaccine involved (Pfizer-BioNTech—Comirnaty, Moderna—mRNA-1273, AstraZeneca—Vaxzevria, and Johnson & Johnson—Janssen).

### Statistical analysis

Continuous variables were described as mean and standard deviation or median and interquartile range as appropriate, categorical variables were expressed as frequencies and percentages. Chi-square test (*χ*^2^) or Fisher exact test, where appropriate, were used to compare the frequency of categorical variables. Differences between groups were analyzed using either the Student *t*-test (parametric variables) or the Mann–Whitney *U*-test (nonparametric). A logistic regression analysis was performed to assess whether specific clinical or demographical characteristics could predict headache etiology. All statistical tests were performed using IBM SPSS 25.0 software for Windows with a statistical significance level set at *p* ≤ 0.05.

## Results

From January 2021 to January 2022, 55,465 patients visited the ED of the Spedali Civili Hospital, of which 2,682 (4.84%) referring headache as their main symptom. Two-hundred and eighty-nine ED headache admissions (10.8% of all ED headache admissions) were time-correlated to the COVID-19 vaccination.

In 2019, the total number of ED presentations for any medical reason was 74,473, of which 3,178 (4.27%) were due to headaches. Thus, the percentage of headache admissions over total ED admissions was higher in 2021 compared with 2019—4.84% vs. 4.27%, respectively (*p* < 0.00001). The difference was significant also when data were analyzed in terms of proportions (*p* < 0.00001). The effect size, given our two sample sizes, was equal to 0.0038. Given that the difference between the two groups was 0.005681, which is bigger than the effect size, we can confirm the difference between the two groups to be statistically significant.

**Figure 1:**
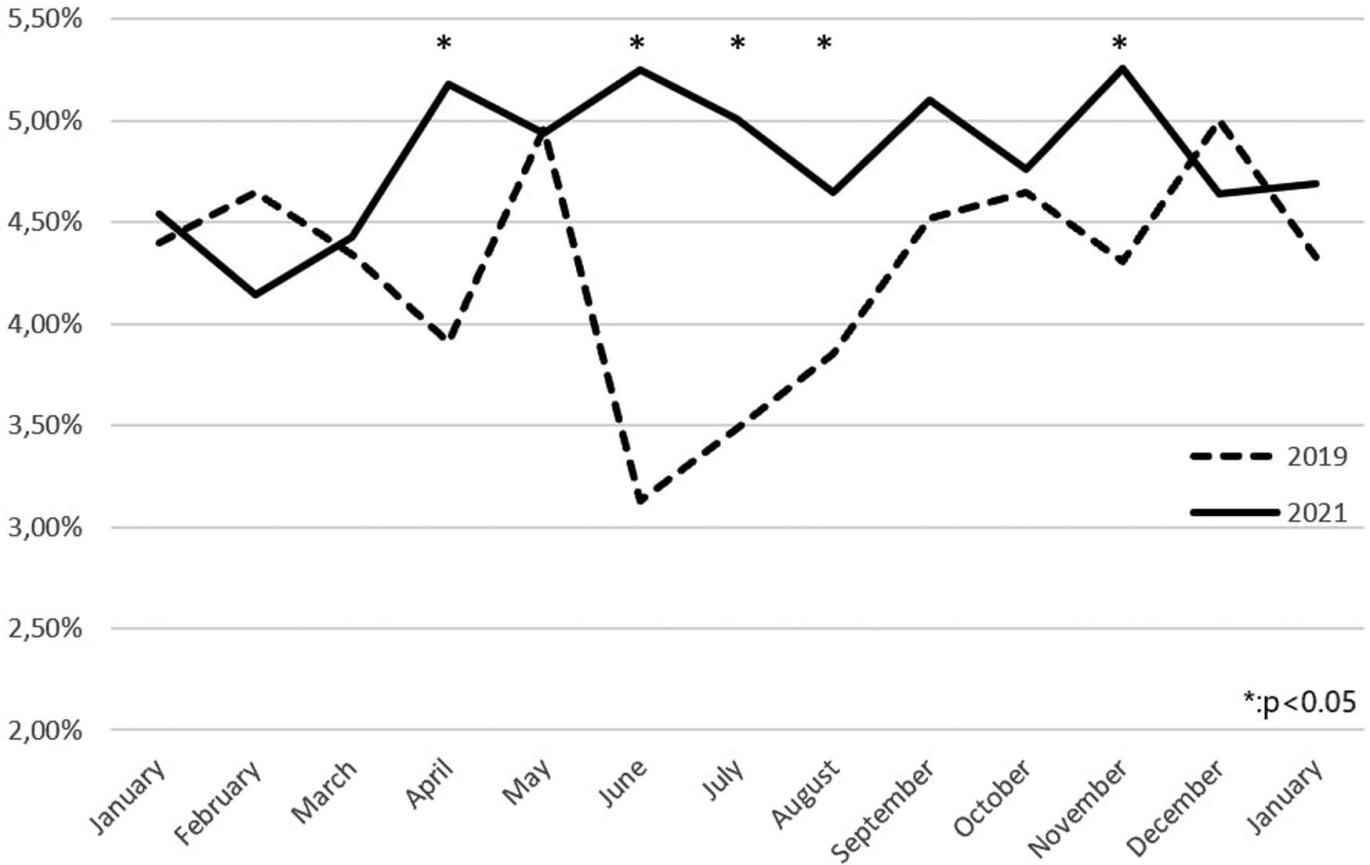
Comparison between emergency department admissions for headache in 2019 and 2021.

Figure 1 shows the percentage of headache admissions over total ED admissions in 2019 and 2021. To note, the highest values in 2021 correspond to those months (June, July, August, and November) in which the vaccination campaign was at its peak (10).

Regarding hospitalizations, in 2021, 152 patients were hospitalized in the Neurology Unit due to headaches, of which 73 (49%) were diagnosed with a secondary headache. In 2019, the number of hospitalized patients due to headaches was 139, of which 81 (58%) were secondary headaches. No statistically significant differences (*p* = 0.08) were found in terms of secondary headaches’ frequencies between 2019 and 2021.

Out of the 289 patients who visited the ED due to headaches time-correlated to COVID-19 vaccination, 40 (13.8%) were hospitalized in order to rule out symptomatic forms. Demographical and clinical characteristics of hospitalized patients are listed in Table 1. All hospitalized patients underwent neuroradiological exams (CT scan, MRI, and/or Magnetic resonance angiography (MRA)). To note, only eight patients reported a pathogenic lesion (see below). The main significant biochemical alteration consisted of D-dimer elevation (ranging from 542 to 5703 ng/ml), reported in six patients. The highest value (5703 ng/ml) was found in a single patient, whose diagnosis was CVT. No patient documented thrombocytopenia. Headache onset was, on average, 5.2 (5.6) days following the vaccination, with a mean duration of 66.4 (78.5) hours. The pain course was described as constantly present in 14/40 patients (35%) and remitting/recurring in 19/40 patients (47.5%). Seven patients (17.5%) referred to headache complete remission at the time of hospitalization. A constant headache was more frequently reported in patients with a secondary headache (75% vs. 25%; *p = 0.02*).

**Table 1:** Demographical and clinical characteristics of all hospitalized patients.

	All patients (*n* = 40)	Benign headache (*n* = 32)	Symptomatic headache (*n* = 8)	*p*
Age; years	45.5 ± 15.6	42.7 ± 13.4	55.3 ± 18.4	NS^a^
(mean ± SD; range)	(17–93)	(17–66)	(39–93)	
Hospitalization; days	5.7 ± 4.4	4.1 ± 1.4	8.0 ± 1.4	*p = 0.003* ^a^
(mean ± SD; range)	(1–19)	(1–19)	(6–13)	
Comorbidity scale (CIRS) score	1.26 ± 0.2	1.29 ± 0.3	1.1 ± 0.1	NS^a^
(mean ± SD; range)	(1.0–1.9)	(1.0–1.9)	(1.0–1.5)	
Systolic blood pressure in ED	125.2 ± 16.1	124.6 ± 17.7	131.4 ± 15.6	NS^a^
(mean ± SD; range)	(90–155)	(90–155)	(106–150)	
Diastolic blood pressure in ED	78.4 ± 12.9	78.03 ± 13.1	78.8 ± 14.7	NS^a^
(mean ± SD; range)	(60–105)	(60–105)	(60–100)	
Body mass index (BMI)	24.2 ± 6.0	23.9 ± 5.2	24.4 ± 7.01	NS^a^
(mean ± SD; range)	(17.9–40.8)	(17.9–40.8)	(18.06–33.05)	
Female gender (frequency, %)	31 (77.5%)	27 (84.3%)	4 (50%)	*p = 0.03* ^b^
History of primary headache (frequency, %)	22 (55%)	20 (62.5%)	2 (25%)	*p = 0.05* ^b^
Family history of primary headache (frequency, %)	13 (32.5%)	9 (28.1%)	4 (50%)	NS^a^
Comorbidities (frequency, %)				
None	8 (20%)	7 (21.8%)	1 (12.5%)	NS^b^
Cardiologic	4 (10%)	2 (6.2%)	2 (25%)	NS^b^
Vascular	1 (2.5%)	0 (0%)	1 (12.5%)	NS^b^
Oncological	3 (7.5%)	3 (9.3%)	0 (0%)	NS^b^
Immunological	1 (2.5%)	1 (3.1%)	0 (0%)	NS^b^
Endocrinological	6 (15%)	5 (15.6%)	1 (12.5%)	NS^b^
Otolaryngology	3 (7.5%)	3 (9.3%)	0 (0%)	NS^b^
Psychiatric	2 (5%)	2 (6.2%)	0 (0%)	NS^b^
Others	14 (35%)	10 (31.2%)	4 (50%)	NS^b^

ED, emergency department.

^a^ Mann–Whitney U-test.

^b^ Chi-square test.

At discharge, 32/40 patients had a diagnosis of benign headache not attributed to any cranial/extracranial disorder and 8/40 patients of secondary headache, whose diagnoses were the following: headache attributed to cranial and/or cervical vascular disorder (one headache attributed to the transient ischemic attack, one headache attributed to nontraumatic intracranial hemorrhage, two headaches attributed to cerebral venous thrombosis); headache attributed to nonvascular intracranial disorder (two headaches attributed to low cerebrospinal fluid pressure); headache or facial pain attributed to disorder of the cranium, neck, eyes, ears, nose, sinuses, teeth, mouth or other facial or cervical structure (one headache attributed to acute rhinosinusitis); and painful lesions of the cranial nerves (one headache attributed to ischemic ocular motor nerve palsy).

Considering the overall number of secondary headaches hospitalized in 2021 (73 patients), the percentage of secondary headaches time-correlated to the COVID-19 vaccination was 10.9% (eight patients).

Patients with symptomatic headaches tended to have significantly longer hospitalizations compared with those with benign headaches (8 ± 1.4 vs. 4.1 ± 1.4; *p* = 0.003). Patients with benign headaches, compared with those with a secondary headache, were more frequently female (84.3% vs. 50%; *p* = 0.03) and with a history of primary headaches (62.5% vs. 25%; *p* = 0.05). Table 2 presents the main headache characteristics (pain features, localization, and intensity; associated symptoms). Motor neurological symptoms were more frequent in patients with symptomatic headaches compared with benign headaches (respectively 25% vs. 0%; *p* = 0.03), whereas nausea and vomiting more frequently accompanied benign headaches (40.6% vs. 0%; *p* = 0.03).

**Table 2:** Headache characteristics of all hospitalized patients.

	All patients (*n* = 40)	Benign headache (*n* = 32)	Symptomatic headache (*n* = 8)	
Pain quality (frequency, %)				
Pulsating	17 (42.5%)	14 (43.7%)	3 (37.5%)	NS^a^
Dull/diffuse	12 (30%)	8 (25%)	4 (50%)	NS^a^
Stabbing	5 (12.5%)	3 (9.3%)	2 (25%)	NS^a^
Pressing	3 (7.5%)	2 (6.2%)	1 (12.5%)	NS^a^
Pain intensity—NRS score (mean, SD)	7.4 (1.7)	7.1 (1.7)	6.75 (1.9)	NS^a^
Pain course during hospitalization (frequency, %)				
Constantly present	14 (35%)	8 (25%)	6 (75%)	0.02^a^
Remitting/recurrent	19 (47.5%)	18 (56.2%)	1 (12.5%)	NS^a^
Resolved within hours of the admission	7 (17.5%)	6 (18.7%)	1 (12.5%)	NS^a^
Localization (frequency, %)				
Frontal	16 (40%)	14 (43.7%)	2 (25%)	NS^a^
Temporal	7 (17.5%)	5 (15.6%)	2 (25%)	NS^a^
Occipital	5 (12.5%)	3 (9.3%)	2 (25%)	NS^a^
Holocranial	8 (20%)	6 (18.7%)	2 (25%)	NS^a^
Unilateral	15 (37.5%)	11 (34.3%)	4 (50%)	NS^a^
Bilateral	22 (55%)	18 (56.2%)	4 (50%)	NS^a^
Neurological associated symptoms (frequency, %)				
Nausea and vomit	13 (32.5%)	13 (40.6%)	0 (0%)	0.03^a^
Photo-phonophobia	9 (22.5%)	8 (25%)	1 (12.5%)	NS^a^
Paraesthesia	8 (20%)	7 (21.8%)	1 (12.5%)	NS^a^
Vertigo	8 (20%)	8 (25%)	0 (0%)	NS^a^
Typical visual aura	4 (10%)	4 (12.5%)	0 (0%)	NS^a^
Motor weakness	2 (5%)	0 (0%)	2 (25%)	0.03^a^
Transient disorders of consciousness	1 (2.5%)	1 (3.1%)	0 (0%)	NS^a^
Diplopia and ptosis	1 (2.5%)	0 (0%)	1 (12.5%)	NS^a^

NRS, numerical rating scale.

^a^ Chi-square test.

However, the logistic regression analysis did not confirm whether the aforementioned features could significantly predict headache etiology.

Overall, the headache most frequently reported by all patients had migraine-like characteristics: the localization was predominantly frontal or temporal, the pain was described as throbbing and severe in intensity and it was frequently accompanied by nausea/vomit, and photo-phonophobia.

Over half (22 patients, 55%) of patients had a history of primary headaches. Indeed, in 20% of the cases, the ED visit and subsequent hospitalization were due to the worsening of a preexisting headache.

Regarding the type of vaccine involved, in our sample, Pfizer-Comirnaty was the predominant (67.5%) among all hospitalized patients, regardless of the discharge diagnosis (primary vs. secondary headache). Spikevax-Moderna accounted for 7.5% and Vaxzevria for the remaining 25% of vaccinations among hospitalized patients.

Headache was reported following the first dose (55% of patients), with the remaining 40% after the second dose and only 5% after the third one. Considering the limited number of secondary headaches and the high Pfizer-Comirnaty predominance, no correlation could be drawn between the type of vaccination and secondary headaches.

## Discussion

In this study, we reported an increased frequency of headache admissions during 2021 compared with 2019, which we hypothesized to be secondary to the high prevalence of headaches correlated to the generic side effects of the COVID-19 vaccination. To support this hypothesis, although the number of ED admissions due to headaches increased in 2021, we did not document an increased number of neurological hospitalizations nor diagnoses of secondary headaches. One might argue that some patients with potential secondary headaches might simply have been referred to other hospitals in the region. However, it is worth stating that the hospital where the present study has been carried out is one of the main structures—in terms of the number of accesses and departments—in the whole region, currently ranking 11th in the World Best Hospital 2021 (11).

The significant increase in ED admissions for headaches might reflect the general population's concern about vaccine-induced side effects, coupled with misinformation concerning vaccines fomented by the news. This, inevitably, had a negative impact on the public health system.

Less than 14% of all the ED visits due to a vaccination time-correlated headache were hospitalized, with most patients documenting a benign headache related to the generic side effects of the vaccination. These headaches would fulfill diagnostic criteria for 8.1 Headache attributed to the use of or exposure to a substance (ICHD-3) (12), although, at the time being, it does not include vaccines as possible substances.

In our cohort of hospitalized patients, secondary headaches were poorly linked to the vaccination, particularly CVTs. In fact, in one patient, the headache was already present before the vaccination and the second patient did not meet the criteria for VITT (13–14). Nonetheless, it cannot be excluded that the vaccination might have played a cofactor in both cases. Moreover, it is worthy of notice that 25% of hospitalized patients with a diagnosis of secondary headaches had a previous history of primary headaches. This finding is crucial as it plays as a reminder for all physicians that pharmacoresistant and/or atypical headaches, also in patients with a previous history of headaches, are worthy of further investigations.

Albeit on a limited cohort, we reported a detailed postvaccination headache profile. Although we could not establish a specific pattern of presentation of secondary headaches due to their limited number, considering all patients, a migraine-like profile came out.

Currently, data regarding postvaccination headache characteristics are scarce. Our study partially confirms the results obtained in a large, multicentric observational study by Göbel et al. (16, 17)—moderate/severe intensity, frontal localization, and association with dysautonomic symptoms (photo-phonophobia, nausea). Similar findings were reported in a recent meta-analysis on headaches post-COVID-19 vaccination (18), which reported a migraine-like pattern of headache in one-third of patients.

The differences regarding the clinical phenotype could be related to the different cohort populations—hospitalized patients vs. the general population receiving the vaccination.

These characteristics could lead to speculations regarding the pathophysiology of the postvaccination headache. It is possible that the immune response elicited by vaccination could activate the trigeminovascular system, resulting in migraine-like headaches in prone individuals. Prostaglandins and bradykinins, released by immune cells, may modulate the release of calcitonin gene-related peptide (CGRP) from neurons and induce migraine attacks (19, 20). Substance P, a nociceptive neuropeptide produced by the trigeminal sensory nerve fibers, is also produced by several immune cells and this could suggest a possible connection between vaccination-induced immune system activation and the subsequent migraine-like headache (16–20).

In our cohort, over half of the hospitalized patients had a history of preexisting primary headaches. It has been reported that a preexisting primary headache such as a migraine was associated with increased duration and pain intensity after COVID-19 vaccination (16, 17, 21). It is possible that the sensitization with hyperexcitability of trigeminovascular neurons, existing in primary headaches, causes an increase in pain sensitivity and frequency.

On the other hand, it has been hypothesized that a role for the SARS-CoV-2 spike-protein is present in all types of COVID-19 vaccines. The spike-protein binds with the ACE2 leading to various biological outcomes, as well as pain modulation. However, it is unknown whether ACE2 is present in peripheral structures of the trigeminal nerve, although some other neuronal structures express ACE2 (22). However, if that was the case, the antibodies developed following the first vaccination dose should significantly reduce the frequency of postvaccination headaches at the second dose, which was not the case both in our study and in others (23).

We acknowledge that our study has several limitations. First, it is a monocentric study with a limited sample—especially regarding secondary headaches, with detailed information available only for hospitalized patients. Second, for the period January to May 2021, the vaccination status was not available for all patients admitted to the ED, thus the precise number of postvaccination headaches could not be inferred, leading to a possible underestimation bias. Third, for patients who spontaneously decided to ED discharge, a secondary headache cannot be excluded.

In conclusion, our findings support the safety of COVID-19 vaccines. Observed postvaccination time-correlated secondary headaches were rare, compared with the overall number of patients with ED visits and hospitalization. Overall, symptomatic post-COVID-19 vaccination headaches accounted for only 0.26% of all ED admissions, with most patients documenting a benign headache possibly related to the generic side effects of the vaccination.

A migraine-like clinical phenotype was characterized, regardless of headache class (primary vs. secondary) or vaccine type.

Further studies are needed to assess the pathogenesis of benign vaccine-induced headaches vs. more threatening secondary headaches, regarding not only the COVID-19 vaccination but all vaccines in general. It would be interesting to assess whether the COVID-19 vaccine was particularly associated with a postvaccination headache compared with other vaccines—assessed in a real-life setting—and whether different types of vaccines (viral DNA vs. recombinant technology) might be particularly associated with headache onset.

Moreover, it is necessary to call for an update of the present classification system, given the prevalence and general magnitude of this type of headache, including vaccines as one of the substances reported in section 8.1 Headache attributed to the use of or exposure to a substance (ICHD-3) (12).

## Data Availability

The raw data supporting the conclusions of this article will be made available by the authors, without undue reservation.
